# Pancreatic mediastinal pseudocyst presenting as a posterior mediastinal mass with recurrent pleural effusions: a case report and review of the literature

**DOI:** 10.1186/s13256-015-0582-z

**Published:** 2015-05-12

**Authors:** Vasilis Karamouzos, Dimitrios Karavias, Dimitrios Siagris, Christina Kalogeropoulou, Fay Kosmopoulou, Charalampos Gogos, Dimitrios Velissaris

**Affiliations:** 1 Internal Medicine Department, University Hospital of Patras, Rion, 26500 Greece; 2 Radiology Department, University Hospital of Patras, Rion, 26500 Greece; 3 General Surgery Department, University Hospital of Patras, Rion, 26500 Greece

**Keywords:** Pancreatic pseudocysts, Mediastinal pseudocysts, Pancreatitis, Alcohol abuse, Recurrent pleuritis, Posterior mediastinal mass

## Abstract

**Introduction:**

A rare complication of chronic pancreatitis is the formation of single or multiple mediastinal pseudocysts, which are fueled from the pancreas through anatomical openings of the diaphragm. We present a rare case with a difficult diagnosis, treatment and potentially catastrophic complications.

**Case presentation:**

A 53-year-old Caucasian man was referred to our hospital for further investigation and treatment of a large heterogeneous mass situated in the posterior mediastinum, and bilateral pleural effusions which had developed after recent multiple episodes of pancreatitis. He had a history of chronic alcoholism. Laboratory and imaging modalities established the diagnosis of a pancreatic mediastinal pseudocyst.

**Conclusions:**

Despite successful initial conservative treatment, our patient had a relapse and underwent emergency surgical intervention due to internal hemorrhage. We present his diagnostic and imaging workup, along with the multidisciplinary intervention, and a literature review referring to the diagnosis and treatment of mediastinal pancreatic pseudocysts.

## Introduction

One of the most common complications of chronic pancreatitis is the development of pseudocysts due to stenosis or disruption of the pancreatic duct. These can be numerous and their size varies from a few centimeters to 30cm in some cases. They are rich in enzymes and are held together by fibrous and granulomatous tissue, hence the term pseudocyst. In some cases they can invade adjacent organs, such as the liver [1,2] and spleen [3], and sometimes may extend above the diaphragm.

We herein present a patient with a history of alcohol abuse who was referred to our hospital for further investigation and treatment of a mass in the posterior mediastinum, and bilateral pleural effusions, which had developed after recent multiple episodes of pancreatitis.

## Case presentation

**Figure 1:**
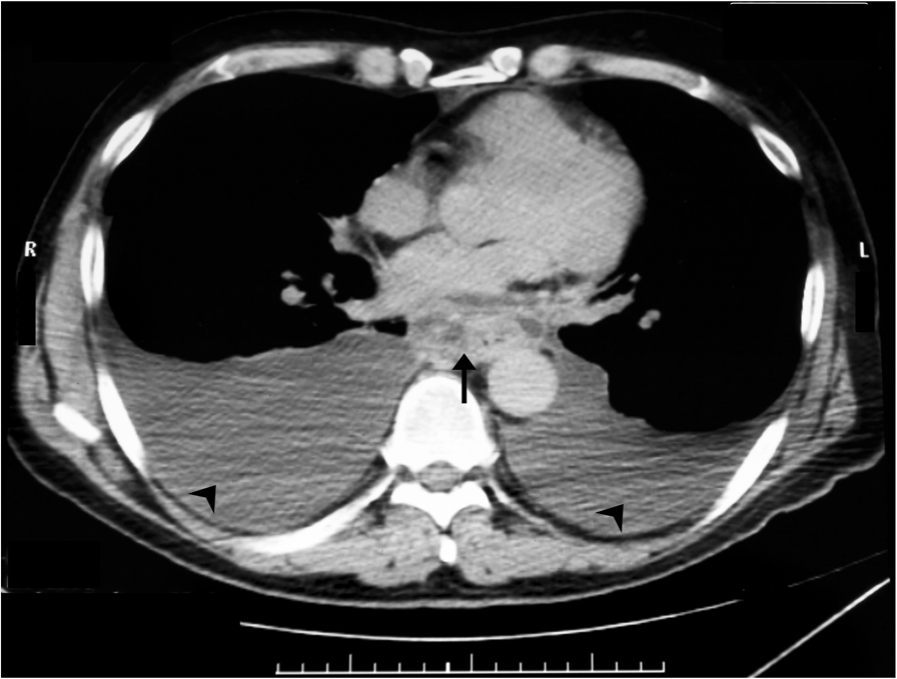
Axial post-contrast computed tomography scan at the level of the lower mediastinum reveals a mass (arrow) and bilateral pleural effusions (arrow heads).

**Figure 2:**
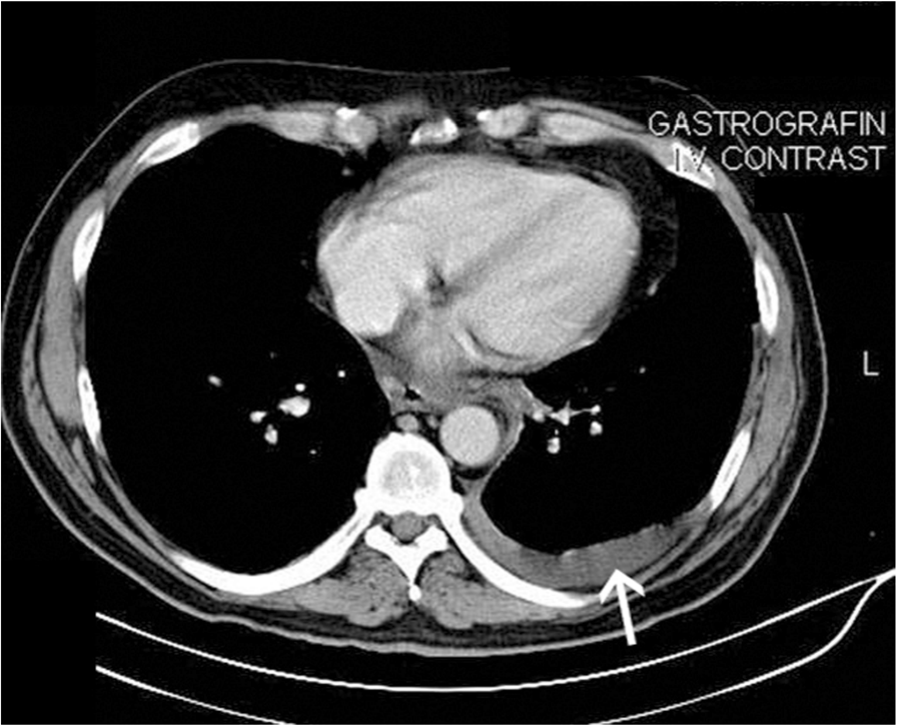
Nine months earlier, an axial post-contrast computed tomography scan reveals no abnormality in the mediastinum. A small pleural effusion is present at the left hemithorax (arrow).

**Figure 3:**
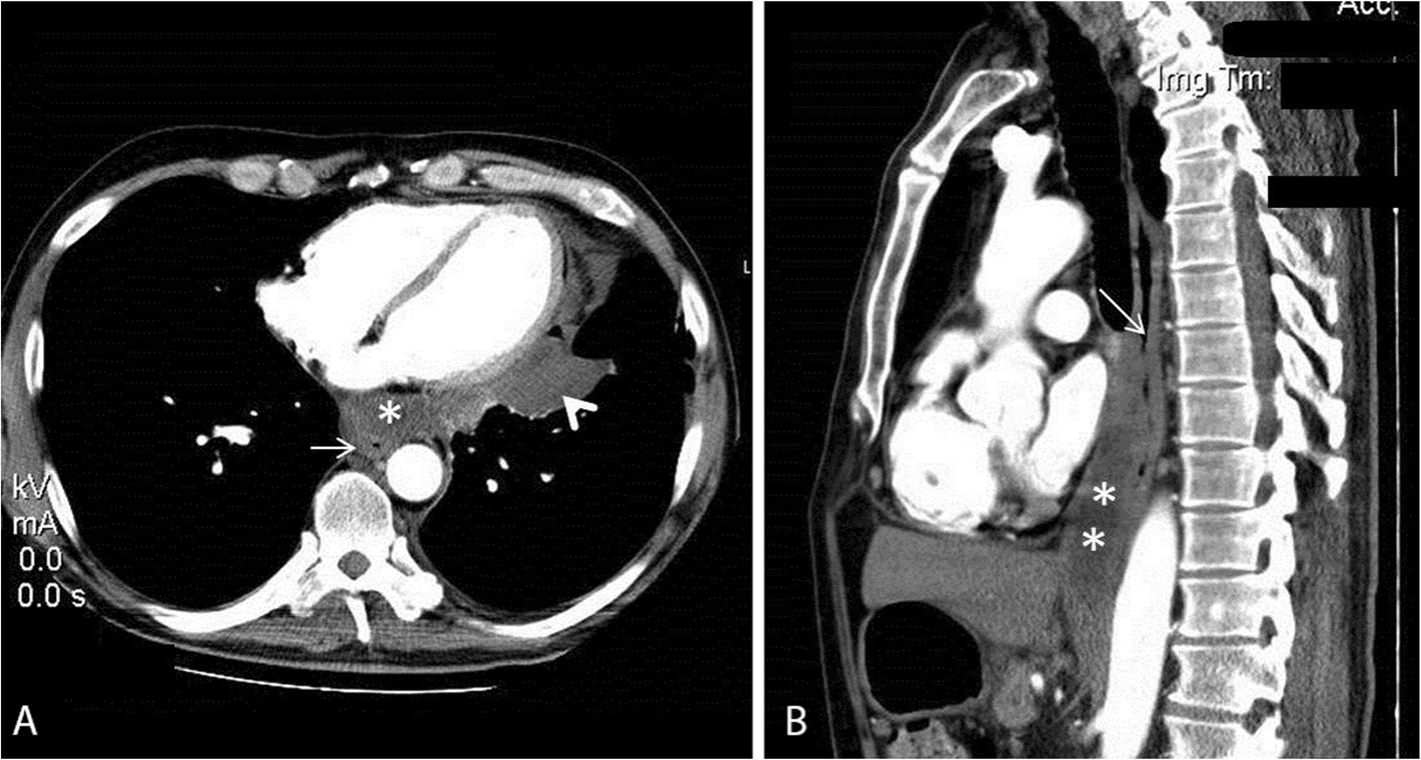
Post-contrast chest computed tomography scan after successful pleural drainage. Panel **A**: Fluid collection (*) in front of esophagus (arrow) and a small pleural effusion is present (arrow head). Panel **B**: Sagittal reconstructed images demonstrate a longitudinal fluid collection (* *) in the mediastinum, along the lower esophagus (arrow), measuring 3×8cm. The lesion was compatible with a mediastinum pseudocyst.

**Figure 4:**
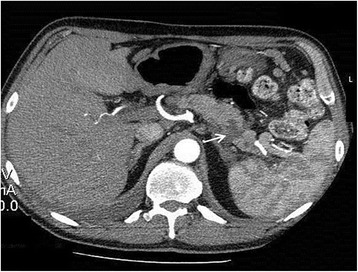
Post-contrast computed tomography scan of the upper abdomen reveals a small pseudocyst of 2cm at the pancreatic tail (arrow).

A 53-year-old Caucasian man with a past medical history of recurrent pancreatitis for the last 18 months, depression, alcohol and tobacco abuse, was referred to our hospital after a 10-day hospitalization in a pulmonary clinic, where he was admitted due to dyspnea and two episodes of hemoptysis. An extensive workup had already been performed, including bronchoscopy, gastroscopy, chest and upper abdomen computed tomography (CT) scans. A diagnostic paracentesis had already been carried out and fluid samples were sent for further analysis. His upper gastrointestinal endoscopy and bronchoscopy did not show any remarkable findings, and his pleural fluid was plenteous in protein and amylase. His cytology reports were negative for malignancy and his pleural fluid adenosine deaminase levels showed no evidence of mycobacterial infection (Table 1). He had a good clinical status, except for a mild shortness of breath on exertion. He reported no fever or chills and, despite having a good appetite the last six months, he noted a weight loss of 25kg. Additionally, he mentioned that over the last four months he had experienced difficulty in swallowing solid food. On admission, his physical examination revealed bilateral diminished breath sounds at the lung basis and a firm liver edge 5cm below the right costal margin. His laboratory results showed anemia, malnutrition and slightly elevated serum amylase (360U/L) (Table 1). His chest CT scan revealed bilateral pleural effusions and a mass in the lower mediastinum (Figure 1); these radiological findings were not present on his previous examinations nine months ago (Figure 2). After a successful chest drainage clinical improvement was noticed, however, further chest CT imaging showed a longitudinal fluid collection in the mediastinum, along the lower esophagus, measuring 3×8cm (Figure 3A, B). This collection extended beyond the diaphragm and seemed to be connected with a small pseudocyst at the pancreatic tail (Figure 4). He was on a specific enteral diet, lipase supplementation and octreotide (administered subcutaneously). Further evaluation with magnetic resonance cholangiopancreatography showed the pancreatic duct to be slightly dilated. No further interventions were performed at that time. A month later he was discharged in good clinical status and scheduled for outpatient observation.

**Table 1:** **Biochemistry**

**Peripheral blood laboratory results**				**Pleural fluid results**	
WBC	4,88K/μL	Ca	8mg/dL	cells	1600
Hb	9.8g/dL	Mg	1.5mg/dL	RBC	224.000
PLT	246K/μL	SGOT	24U/L	TP	4.8gr/dL
PT	14.5sec	SGPT	44U/L	ALB	2.8gr/dL
APTT	35.6sec	ALB	3.1gr/dL	GLU	82mg/dL
D-Dimers	6.810md/dL	TGL	80mg/dL	LDH	609U/L
FIB	521μg/dL	AMS	360U/L	AMS	12,881U/L

WBC, white blood cells; Hb, hemoglobin; PLT, platelets; PT, prothrombin time; APTT, activated partial thromboplastin time; FIB, fibrinogen; Ca, Calcium; Mg, Magnesium; SGOT, serum glutamic oxaloacetic transaminase; SGPT, serum glutamate-pyruvate transaminase; ABL, albumin; TGL, triglycerides; AMS, amylase; RBC, red blood cells; TP, total protein; GLU, glucose; LDH, lactate dehydrogenase.

Two months after his discharge he was readmitted with clinical signs of respiratory distress and bilateral pleural effusion was visible on his radiology chest imaging. The results from his serum blood and pleural fluid laboratory tests were similar to his first admission. As similar findings were identified on his chest and abdomen CT scan compared to the images from his last admission, a decision for surgical intervention was made. Upon completion of the preoperative diagnostic workup he was transferred to the general surgery ward. He was on a triple antibiotic regimen (meropenem, vancomycin and fluconazole) and was scheduled for elective surgery. On the fourth hospitalization day he suffered from nausea, vomiting and abdominal pain. His physical exam showed that his abdomen was rigid, with noted pain on the upper abdomen and guarding. His laboratory tests showed a significant drop in his hemoglobin level and his vital signs revealed borderline tachycardia. After resuscitation with fluids and blood transfusions, a CT scan of his abdomen was performed where an extended hematoma at the lesser sac was identified with no active bleeding. Subsequently, he underwent an elective embolization of the splenic artery followed by an emergency laparotomy. Intraoperatively, a sizable hematoma was found at the lesser sac, pushing his stomach anteriorly. Due to chronic inflammation his pancreas was fibrous and hard. The splenic vein was thrombosed and splenomegaly with left-sided portal hypertension was present. A distal pancreatectomy with splenectomy was performed; his post-operative course was uneventful and he was discharged on the 11^th^ postoperative day.

## Discussion

Tumors that are most commonly located in the posterior mediastinum are of esophageal and nervous origin. Hernias, aortic aneurysms and duplication cysts can also be found in this anatomic area. Some cases of extramedullary hematopoiesis [4] and pancreatic pseudocysts are also reported in the literature. After a rupture of the posterior pancreatic duct, the pancreatic fluid is usually confined in the retroperitoneal space, but can occasionally slip into the posterior mediastinum from the esophageal or the aortic hiatus, leading to the formation of pseudocysts. If the diaphragm is penetrated through the inferior vena cava hiatus or the foramen of Morgagni, the pseudocyst will be formed in the middle and anterior mediastinum, respectively [5]. Published data report that these cysts can occur at any age from 7 months to 73 years, and the leading cause in adults is chronic alcoholic pancreatitis and trauma in children [6]. The related symptoms may vary from chest pain, palpitations, dysphagia, pleural effusion with concomitant dyspnea, cough and hemoptysis, to severe life-threatening complications such as hemothorax, cardiac tamponade, esophagobronchial fistula and acute airway obstruction [7-10].

Computed tomography and magnetic resonance imaging are the most commonly used imaging modalities to reveal the size, location and possible communications of the pseudocysts with the pancreas. Furthermore, magnetic resonance computed pancreatography in conjunction with esophageal retrograde cholangiopancreatography can unveil strictures and disruption of the pancreatic duct. A transesophageal fine needle aspiration can be performed when a differential diagnosis problem is raised, but the patient’s medical history and CT imaging study are usually adequate for the diagnosis to be established.

The management of a mediastinal pancreatic pseudocyst is a difficult task. Relapses are common and procedural complications are many. At the moment there are no guidelines addressing this problem. A literature review revealed only a few scant reports of spontaneous resolution [11,12], but in the vast majority of cases intervention was mandatory, and the approach has to be individually tailored.

Conservative treatment with strict diet, enzyme supplementation and complete abstinence from alcohol is indicated to all stable patients. In addition, bromhexine hydrochloride and somatostatin analogues have been used in a few cases [13-15]. Somatostatin and its analog octreotide are peptide hormones that inhibit the release of insulin, glucagon, gastrin, secretin, pancreozymin and pepsin. In our patient, octreotide was successfully administered at a dose of 0.5mg (administered subcutaneously), three times daily, for one month following his first admission.

In certain cases, minimally invasive procedures can resolve the problem and help patients to avoid surgery. Patients with pancreatic duct disruption and stenosis can be treated with the placement of stents or with naso-pancreatic drainage, in order to restore the duct patency and seize the pseudocyst fuelling. For other patients with large abdominal pseudocysts that communicate with the mediastinum, CT-guided abdominal percutaneous drainage may be the optimal treatment [16-20]. In our patient, the magnetic resonance cholangiopancreatography showed no patency problems of the pancreatic duct, and the 2cm cyst beside the pancreatic tail was too small to be accessed and drained. To date, surgery with or without pancreatectomy is the most commonly used treatment, alongside internal drainage of the stomach, or percutaneous drainage when applicable.

## Conclusions

Mediastinal pancreatic pseudocyst is a rare complication of alcohol related pancreatitis, with potentially catastrophic complications and a difficult treatment course. A multidisciplinary approach is mandatory for achieving an optimal outcome.

## Consent

Written informed consent was obtained from the patient for publication of this case report and any accompanying images. A copy of the written consent is available for review by the Editor-in-Chief of this journal.
